# Prognostic significance of Pleural Fluid triglyceride levels based on a low-Fat Diet Management Strategy in patients with Chylothorax following pulmonary resection

**DOI:** 10.1186/s13019-024-02850-4

**Published:** 2024-06-20

**Authors:** Hua Ji, Zhen Wang, Cui Xu, Xiaofeng Yu, Haibo Huang

**Affiliations:** Department of thoracic Surgery, Yantai Yuhuangding Hospital, Medical College of Qingdao University, 20th Yuhuangdingdonglu, YanTai, 264000 ShanDong China

**Keywords:** Chylothorax, Pulmonary resection, Low-fat diet, Triglyceride level, Pleural fluid

## Abstract

**Background:**

Chylothorax is a postoperative complication in patients with lung cancer. Diet-control approaches have been the mainstay for managing this condition. However, a surgical intervention is needed for the patients if conservative treatment is ineffective. Because of the lack of accurate indicators to assess the prognosis of the postoperative complication at an early stage, the criteria of surgical treatment were not consistent.

**Methods:**

We reviewed 2942 patients who underwent pulmonary resection and lymph node dissection for primary lung cancer at our hospital between March 2021 and December 2022. The prognostic implications of clinical indicators were assessed in patients with postoperative chylothorax who were managed with a low-fat diet. Binary logistic regression was used to explore the predictive value of these indicators for patient prognosis.

**Results:**

Postoperative chylothorax occurred in 108 patients and 79 patients were treated with a low-fat diet management while 29 patients were managed with TPN. In contrast to drainage volume, the pleural effusion triglyceride level after 2 days of low-fat diet exhibited enhanced predictive efficacy in predicting patient prognosis. When the pleural fluid triglyceride level of 1.33 mmol/L was used as the diagnostic threshold for prognosis, the sensitivity and specificity reached 100% and 80.6%, respectively.

**Conclusions:**

The pleural effusion triglyceride level after 2 days of low-fat diet can serve as a valuable prognostic indicator in patients undergoing lung surgery and experiencing chylothorax. This predictive approach will help thoracic surgeons to identify patients with poor prognosis in a timely manner and make decision to perform necessary surgical interventions.

## Background

Chylothorax is an uncommon but potentially severe postoperative complication of pulmonary resection and lymph node dissection for lung cancer. It results from injury to the thoracic duct or its tributaries during surgery. Chylothorax is frequently diagnosed based on pleural fluid triglyceride level testing [1]. The mortality rate of untreated chylothorax is high. Continuous leakage of chyle into the pleural space is associated with respiratory symptoms, nutritional deficiency, and immunological morbidity [1]. Unfortunately, there are no established guidelines for managing chylothorax following pulmonary resection.

Dietary management consisting of total parenteral nutrition (TPN) and a low-fat diet has been the mainstay treatment for chylothorax [2]. However, if conservative treatment fails, surgical interventions are often considered depending on the required volume of pleural drainage. Previous studies have reported inconsistent criteria for reoperation, with required drainage volumes ranging from 400 mL/day to 1000 mL/day and reoperation timing varying from the first day to more than 2 weeks after initial surgery [3–9]. Even though Cho et al. considered that air leakage and chest tube output greater than 21.6 mL/kg for 5 days after dietary treatment were significant predictive factors for dietary treatment failure [10], It lacks accurate indicators to assess the prognosis of the postoperative complication at an early stage.

Therefore, we reviewed our low-fat diet management strategy to evaluate and identify more predictors of poor prognosis in patients with lung cancer and postoperative chylothorax. The findings of this study will assist surgeons to distinguish patients with poor prognosis and decide early to perform necessary surgical interventions in this population.

## Patients and methods

### Patients

This study was a retrospective analysis approved by the Yantai Yuhuangding Hospital Research Ethics Committee (Approval No. 2023 − 381), and the need for individual informed consent was waived.

The subjects of this study included patients who were diagnosed with chylothorax after pulmonary resection and lymph node dissection for primary lung cancer between March 2021 and December 2022 because that during this period, a low-fat diet management strategy was implemented for this population at our department. Patients who were not treated with this diet strategy were excluded from the part of analysis for reliable predictors in our study.

### Diagnosis of Chylothorax and Management

Following a normal diet, postoperative patients who presented with white or turbid drainage in their chest tubes underwent pleural fluid triglyceride level testing. If the test showed triglyceride levels above 1.24 mmol/L(> 110 mg/dL), chylothorax was diagnosed [1]. Pleural fluid triglyceride levels were determined using the GPO-POD assay kit (Beckman Coulter, Suzhou, China).

From March 2021, most of the patients with chylothorax in our department were managed with a low-fat diet, which restricted fat intake(≤ 5 g/day). This dietary regimen was closely supervised by nutritionists at our hospital who prohibited the consumption of any food or drinks containing fat. However, some surgeons in our department have advocated for complete cessation of oral intake with TPN as a management strategy for patients with postoperative chylothorax. There was not a detailed description of these patients in this study because we did focus on the low-fat diet management strategy.

**Fig. 1:**
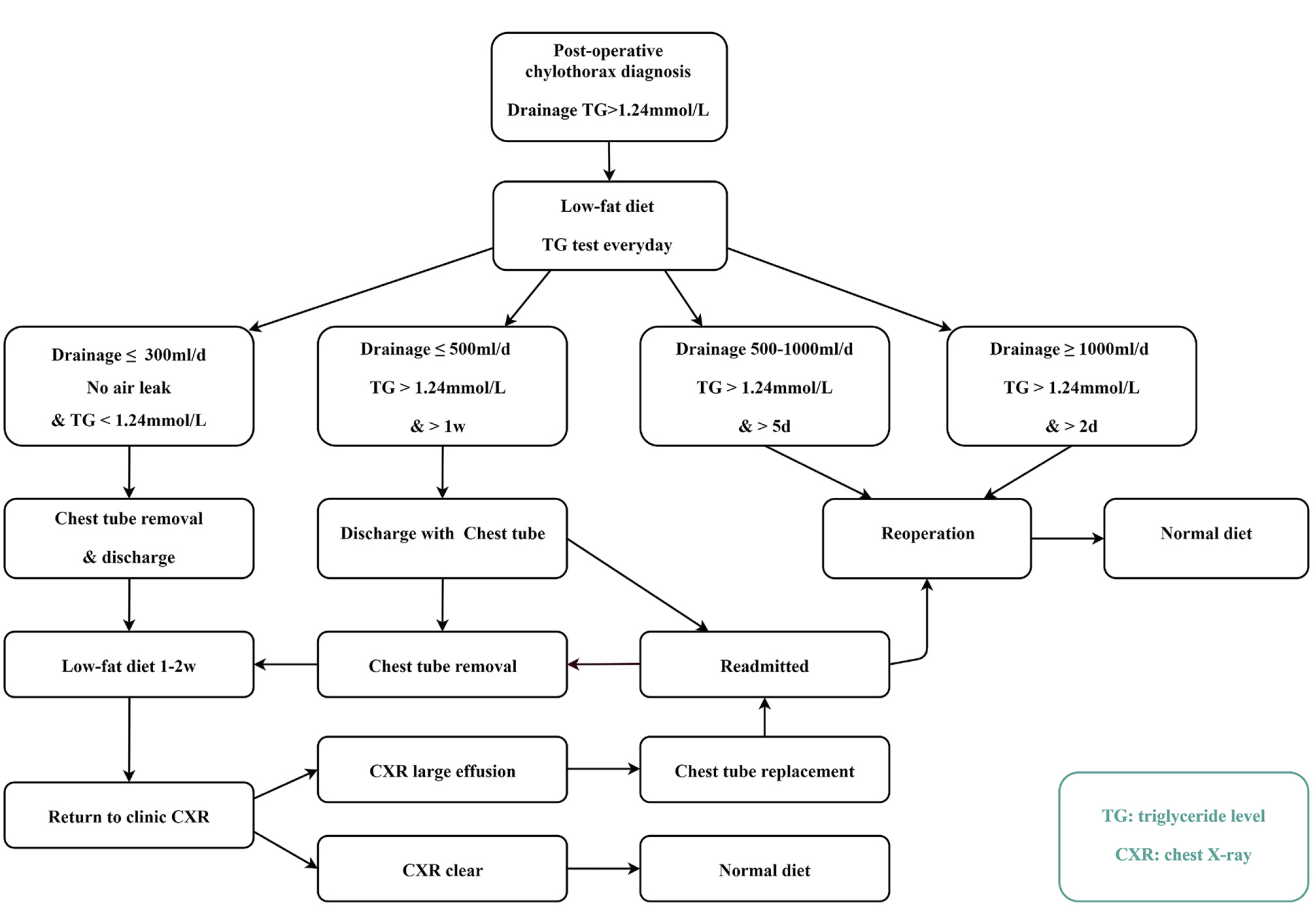
Flowchart of the chylothorax management strategy in this study

In this study, the low-fat diet management strategy for chylothorax is shown in Fig. 1. The amount of drainage fluid was recorded daily, and pleural fluid triglyceride (TG) levels were tested from chest tubes as an indicator of chylothorax. The criteria for intervention were based on daily triglyceride level until one of the following conditions was met. If the triglyceride level was less than 0.56 mmol/L, indicating a low probability (less than 5%) of chylous fluid [1], or if the chest tube was removed, or before reoperation or within the initial 7 days for patients experiencing prolonged chyle leakage. Pleurodesis was performed through their chest tubes in patients with air leakage.

Patients were managed using distinct strategies depending on the amount of drainage and TG levels. To be more specific, (1) Resolution strategy: when the chest tube output became clear, with no air leakage and fluid less than 300 mL/day and TG levels less than 1.24mmpl/L, the tube was removed. Patients was discharged with instructions to continue a low-fat diet for 1 or 2 weeks. A chest radiograph was performed on the last day of the dietary intervention. The patients were instructed to switch to a normal diet if the results were normal. In case of a new large effusion, the patient was readmitted, and a chest tube was placed. (2) Intermediate strategy: If drainage ranged between 300 and 500 ml/day with TG levels higher than 1.24mmol/L for more than one week, patients could be discharged with the chest tube in place. Patients continue the low-fat diet for 1week, then return to our clinic. Chest tube removal followed the same criteria as the Resolution strategy. However, if removal criteria were not met, patients might be readmitted. (3) Reoperation criteria: reoperation was considered necessary when the drainage exceeded 500 ml/day for more than five days, or exceeded 1000 ml/day for more than two days, with TD levels higher than 1.24mmol/L.

### Data collection and definition

The clinical characteristics of patients were recorded for subsequent analyses. These characteristics included triglyceride level after 2 days of low-fat diet and 11 additional perioperative variables: sex, age, drainage volume, operation side and type, number of lymph node dissection stations (N1 stations and N2 stations), postoperative serum albumin, hemoglobin, platelet count, lymphocyte percentage, and lymphocyte count. We selected triglyceride levels after 2 days of low-fat diet for two reasons: to eliminate the influence of normal dietary intake when chylothorax was observed, and some patients’ chest tubes were removed on the third day.

In this retrospective study, patients were stratified into two groups: a group with good prognosis and a group with poor prognosis. Although there is no unique threshold for defining a good prognosis chyle leak, conservative treatment should not be attempted for more than 2 weeks [11]. Therefore, in this study, we defined the good prognosis group as patients who required chest drainage for two or fewer weeks; while the poor prognosis group included patients who required chest drainage for more than 2 weeks, patients who were readmitted with prolonged or recurrent chylothorax, and patients who underwent reoperation.

### Statistical analysis

The distribution of basic characteristics between the two groups was assessed using independent sample t-tests or Pearson’s chi-square tests. Significant variables or those that impacted prognosis were included in the binary logistic regression analysis to identify reliable predictors. Additionally, no correlation was allowed between the variables included in the logistic regression analysis. Subsequently, multivariate logistic regression analysis was performed to identify variables that could independently predict prognosis.

The prediction model is evaluated using a receiver operating characteristic (ROC) curve. A decision curve analysis (DCA) was performed to assess the practical value of the model. The flow of analysis was completed using the pROC and rmda packages in R.

IBM SPSS Statistics (version 24.0) and R version 4.2.1 software were used for statistical analyses. Significance was set at *p* < 0.05.

## Results

### Patient characteristics

From March 2021 to December 2022, 2942 patients with lung cancer underwent surgical treatment by video-assisted thoracoscopic surgery (VATS) and robot-assisted thoracic surgery (RATS) in our department; of these, 108 patients had postoperative chylothorax. The incidence of chylothorax after surgery in patients with lung cancer was approximately 3.67%.

Among all 108 patients, 29 patients received TPN and 79 patients undergone a low-fat diet management. Assessment of prognosis revealed a 20.7% (6/29) incidence of the poor group in the patients with TPN management, while the low-fat diet group exhibited a 15.2% (12/79) occurrence. But statistical analysis indicated no significant difference between the two groups (*P* = 0.497).

**Fig. 2:**
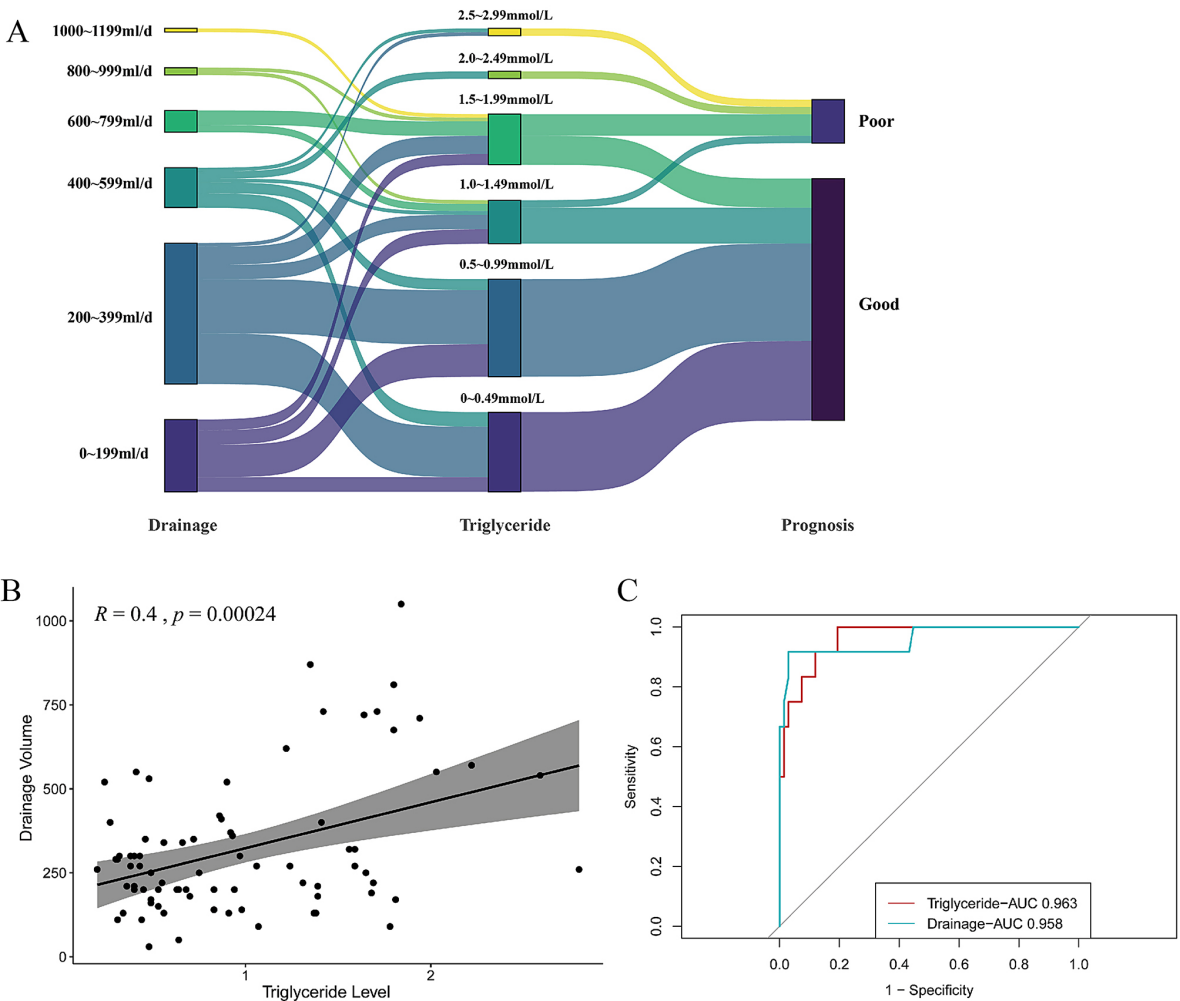
(**A**) A Sankey plot showing the correlation between triglyceride levels, drainage volumes, and prognosis of patients with chylothorax. (**B**) Correlation between drainage volume and triglyceride levels. (**C**) The receiver operating characteristic (ROC)curve of the two models by logistic regression

Among the 79 patients receiving a low-fat diet management, 67 in the good prognosis group exhibited clinical improvement within 2 weeks of initiating a low-fat diet, whereas the remaining patients exhibited a poor prognosis. The relationship between triglyceride levels, drainage volumes, and prognosis of patients with chylothorax was visually represented using a Sankey plot (Fig. 2A).

Of the 12 patients with a poor prognosis, 4 patients were with periods of indwelling chest tubes (18–42 days), and 8 underwent reoperation. During surgery, lymphatic fistulas were identified and found to be localized in lymph node stations 4R (6 cases), 2R (1 case), and 7 (1 case). Pleurodesis was carried out for 3 patients and 4 patients in the good and the poor group respectively. All patients were followed up for 6months. None of the patients in either group died. No patient experienced chylothorax recurrence after reoperation.

**Predictors for prognosis of patients with chylothorax.**

This study collected various characteristics for binary logistic regression analysis, including demographic variables such as sex and age as well as clinical variables such as triglyceride level and drainage volume after 2 days receiving a low-fat diet. Other variables included the operation side and postoperative laboratory values such as serum albumin, hemoglobin, platelets, lymphocyte percentage, and lymphocyte count.

Table 1 presents the characteristics of patients with different prognosis. The triglyceride levels and drainage volumes after receiving a low-fat diet were significantly different between the two groups (*p* < 0.05). However, triglyceride levels and drainage volumes were correlated making it impossible to include both variables in the binary logistic regression (*R* = 0.4, *p* = 0.00024,Fig. 2B). Therefore, we conducted separate binary logistic regressions using triglyceride levels and drainage volumes to identify effective predictors for the prognosis of chylothorax patients. Considering that age and operation side are important clinical factors, we included them in the logistic regression analysis. The results of the multivariate logistic regression analysis are presented in Table 2. Based on these results, triglyceride levels and drainage volumes are independent risk factors for the prognosis of patients with chylothorax. However, triglyceride levels had a more advantageous area under the ROC curve than drainage volume (0.963 vs. 0.958, Fig. 2C).

**Table 1:** Comparison of clinical characteristics between the two groups

	Good prognosis group (*N* = 67)	Poor prognosis group (*N* = 12)	*P*-value
**Sex**			
Female	42 (62.7%)	8 (66.7%)	1
Male	25 (37.3%)	4 (33.3%)	
**Age**			
Mean (SD)	58.6 (10.1)	60.0 (8.99)	0.643
Median [IQR]	59.0 [52.0, 66.0]	60.5 [52.0, 68.0]	
**Operation side**			
Left	16 (23.9%)	1 (8.3%)	0.409
Right	51 (76.1%)	11 (91.7%)	
**Operation type**			
Lobectomy	45 (67.2%)	7 (58.3%)	0.792
Sublobectomy	22 (32.8%)	5 (41.7%)	
**Dissected N1 stations**			
Mean (SD)	3.09 (1.11)	2.75 (0.866)	0.248
Median [Min, Max]	3.00 [0, 6.00]	3.00 [0, 3.00]	
**Dissected N2 stations**			
Mean (SD)	2.28 (1.23)	2.00 (1.48)	0.541
Median [Min, Max]	3.00 [0, 4.00]	3.00 [0, 3.00]	
**Triglyceride level after 2 days of low-fat diet**			
Mean (SD)	0.805 (0.455)	1.93 (0.432)	<0.001
Median [Min, Max]	0.660 [0.200, 1.81]	1.82 [1.35, 2.80]	
**Drainage**			
Mean (SD)	255 (122)	685 (196)	< 0.001
Median [Min, Max]	220 [30.0, 620]	715 [260, 1050]	
**Albumin**			
Mean (SD)	36.4 (5.80)	37.4 (6.89)	0.629
Median [Min, Max]	35.6 [28.5, 58.7]	35.9 [28.3, 53.5]	
Missing	17 (25.4%)	0 (0%)	
**Platelet**			
Mean (SD)	213 (58.3)	202 (41.6)	0.431
Median [Min, Max]	202 [25.9, 392]	209 [134, 259]	
**Hemoglobin**			
Mean (SD)	4.34 (0.539)	4.41 (0.530)	0.683
Median [Min, Max]	4.33 [3.04, 5.69]	4.34 [3.64, 5.46]	
**Lymphocyte percentage**			
Mean (SD)	12.1 (5.85)	14.1 (6.07)	0.298
Median [Min, Max]	10.3 [3.60, 27.9]	12.0 [7.40, 25.6]	
**Lymphocyte count**			
Mean (SD)	1.17 (0.372)	1.14 (0.485)	0.821
Median [Min, Max]	1.13 [0.510, 2.18]	1.09 [0.630, 2.26]	

**Table 2:** Multivariate logistic regression analysis of two models predicting the prognosis of patients with chylothorax

	Variables	β	OR	95% CI	*p* value
**Model**	Age	-0.031	0.970	0.869–1.082	0.580
	Operation side				
	Left		1		
	Right	-0.837	0.433	0.026–7.174	0.559
	Triglyceride	6.530	685.626	7.558–6.22e + 04	0.005
**Model**	Age	0.075	1.078	0.943–1.232	0.273
	Operation side				
	Left		1		
	Right	-1.809	0.164	0.006–4.587	0.287
	Drainage	0.016	1.017	1.007–1.026	0.001

CI, confidence interval; OR, odds ratio

### Establishment of the prediction model

**Fig. 3:**
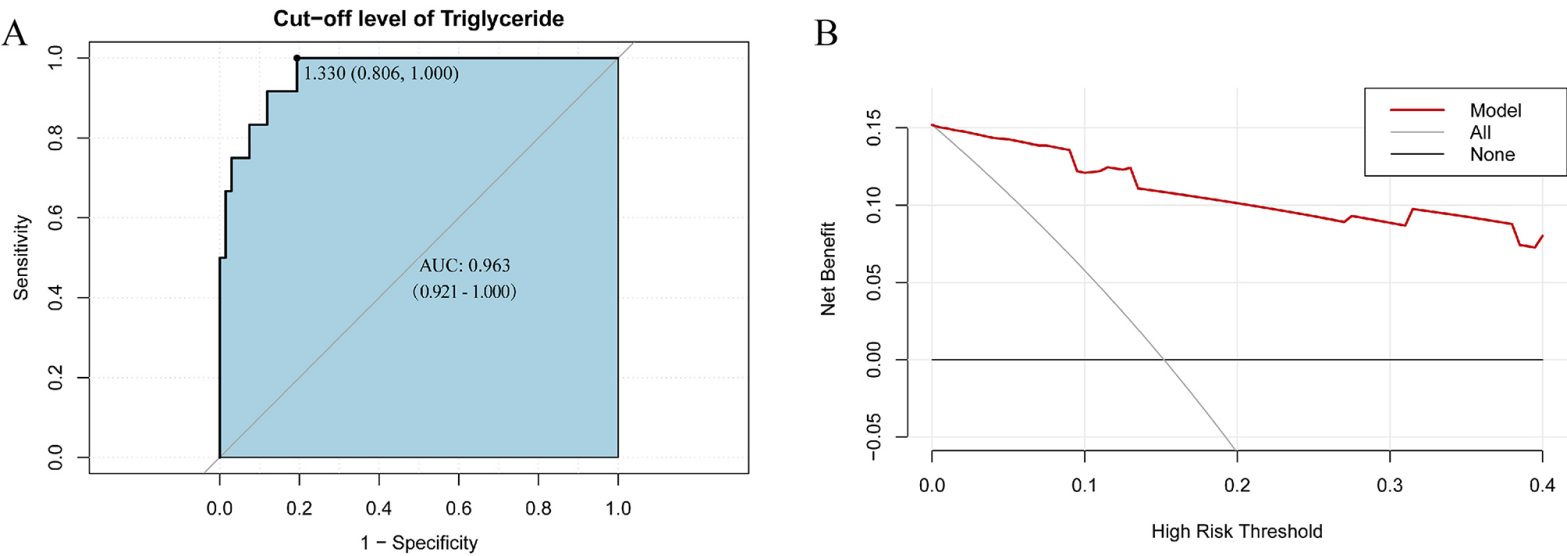
(**A**) The ROC curve of the model and the cut-off value of triglyceride level. (**B**) Decision curve of the prediction model

Binary logistic regression analysis was conducted to investigate the relationship between triglyceride levels and specific outcomes. Based on these results, a predictive model was developed using triglyceride level as the predictor variable. The accuracy of the model was evaluated using an ROC curve, which showed an area under the curve (AUC) of 0.963 (0.921–1.000), indicating high predictive ability (Fig. 3A). ROC curve analysis indicated that the critical value for triglyceride level for predicting the prognosis of patients with chylothorax was 1.33, with a sensitivity of 100% and specificity of 80.6%.

The DCA results (Fig. 3B) showed a good net benefit, suggesting that the predictive model could provide effective assistance for clinical decision making. These findings demonstrate that triglyceride level is a valuable predictor of the outcome of interest, and the developed predictive model could potentially improve clinical decision making in this area.

## Discussion

Chylothorax is not rare following pulmonary resection for lung cancer [3]. Our findings demonstrated an incidence rate of 3.67%, which aligns with the findings of Uchida et al., who reported a rate of 4%, while previous studies have reported rates of approximately 2% [3, 6–10, 12]. Notably, mediastinal lymph node dissection has consistently emerged as a significant contributor to the increased occurrence of chylothorax across various studies. In our investigation of reoperation patients, we identified the 4R lymph node station as the primary site of chyle leak, especially from the small lymphatic collaterals in this region. These findings highlight the need for caution when dissecting the mediastinal lymph node to minimize the risk of chylothorax in patients undergoing pulmonary resection for lung cancer.

TPN has traditionally been employed as a treatment strategy for postoperative chylothorax in patients with lung cancer [3, 4, 6, 8, 10]. However, recent studies have proposed a low-fat diet as an effective alternative, offering comparable outcomes while avoiding complications associated with complete oral intake cessation and reducing patient costs [10, 12]. In our study, there was no statistically significant difference between the two management strategies regarding the assessment of prognosis. Yasuura et al. conducted a study using a low-fat diet as a management approach for postoperative chylothorax, resulting in relief for 95% of patients [9]. Similarly, in this study, 89.8% of patients with postoperative chylothorax experienced resolution of their condition with a low-fat diet. These findings indicate that a low-fat diet may serve as a viable treatment option for postoperative chylothorax in patients with lung cancer, providing comparable efficacy to TPN while potentially mitigating the associated risks and reducing costs. Additionally, fasting can lower triglyceride levels, and the changes in triglyceride levels in the chylous may not accurately reflect chylothorax improvement [13]. For this reason, we did not test the pleural fluid triglyceride levels for patients who were treated with TPN.

In this study, patients with poor prognosis experienced extended periods of indwelling chest tubes (18–42 days) or reoperation. The aim of our study was to examine the initial clinical characteristics of this patient group. It is widely recognized that a decrease in drainage volume serves as a predictive indicator of postoperative improvement of chylothorax. Previous studies have also identified postoperative drainage volume as a determinant for assessing the necessity of surgical intervention. However, the reported reference values for drainage volume in different studies vary markedly, ranging from > 400 mL/day to ≥ 1000 mL/day.^3–9^In postoperative chylothorax patients with lung cancer, the pleural fluid comprises both exudative chylous and transudative nonchylous effluent [13]. It was safe to remove a chest tube with nonchylous drainage of 450mL/d after pulmonary resection [14]. Therefore, to some extent, using drainage volume as an indicator for reoperation is not accurate. In our study, when comparing the predictive efficacy of drainage volume and pleural fluid triglyceride levels in predicting patient prognosis, the latter demonstrated superior performance.

We evaluated whether postoperative chylothorax was improved by measuring triglyceride levels in the pleural fluid. This method has been commonly employed to diagnose and rule out chylothorax [1]. The primary function of the thoracic duct and its branches is to transport digested fat to the venous system. Consequently, a sustained reduction in pleural fluid triglyceride levels achieved by maintaining a consistent low-fat diet may indicate natural healing of damaged lymphatic vessels. In our study, when a pleural fluid triglyceride level of 1.33 mmol/L was used as the diagnostic threshold for prognosis, the sensitivity and specificity reached 100% and 80.6%, respectively. These results indicate that when the patient’s pleural fluid triglyceride level is more than 1.33 mmol/L, the likelihood of a poor prognosis is 100%.

## Limitation

It is important to acknowledge the limitations of the present study. First, the sample size used to construct the prediction model was relatively modest despite the fact that the number of patients included in the present investigation exceeded that of previous studies focusing on postoperative chylothorax in lung cancer. Furthermore, this study adopted a retrospective design within a single tertiary hospital situated in Shandong, China. Hence, future large sample size and multicenter prospective trials are necessary to validate this risk prediction model.

## Conclusions

Based on these findings, we posit that after a low-fat diet intervention, early assessment of pleural fluid triglyceride levels can serve as a valuable prognostic indicator for patients undergoing lung surgery and with chylothorax. This predictive approach has the potential to provide effective guidance in clinical practice and will help thoracic surgeons when deciding to perform surgical interventions in this population.

## Data Availability

No datasets were generated or analysed during the current study.

## Abbreviations

AUC: Area under the curve
TPN: Total parenteral nutrition
ROC curve: Receiver operating characteristic curve
DCA: Decision curve analysis
VATS: Video-assisted thoracoscopic surgery
RATS: Robot-assisted thoracic surgery
TG: Triglyceride
